# Prevalence of the potentially inappropriate Kampo medications to be used with caution among elderly patients taking any prescribed Kampo medications at a single centre in Japan: a retrospective cross-sectional study

**DOI:** 10.1186/s12906-018-2228-3

**Published:** 2018-05-11

**Authors:** Junpei Komagamine, Kazuhiko Hagane

**Affiliations:** 1 Department of Internal Medicine, National Hospital Organization Tochigi Medical Center, 1-10-37, Nakatomatsuri, Utsunomiya, Tochigi 3208580 Japan; 2 Department of Paediatric Surgery, National Hospital Organization Tochigi Medical Center, 1-10-37, Nakatomatsuri, Utsunomiya, Tochigi 3208580 Japan

**Keywords:** Elderly, Kampo, Japanese herbal medicine, Potentially inappropriate medication

## Abstract

**Background:**

Few studies have investigated the prevalence and characteristics of potentially inappropriate Kampo medication use among elderly ambulatory patients taking any prescribed Kampo medications.

**Methods:**

A retrospective cross-sectional study was conducted using electronic medical records. All patients aged 65 years or older who continued visiting internal medicine physicians and were prescribed any Kampo medications from January 2015 to March 2015 were included. The primary outcome was the proportion of patients taking any potentially inappropriate Kampo medications that should be used with caution (hereafter referred to as UWC Kampo medications). The medication appropriateness was evaluated based on the 2015 Japan Geriatrics Society guidelines.

**Results:**

Eighty eligible patients were identified. The mean age was 76.7 years, 45 patients (56.2%) were female, the mean Charlson Comorbidity Index was 1.7, the median number of non-Kampo medications used was 5.0, and the median number of Kampo medications used was 1.0. The proportion of patients taking any UWC Kampo medications was 28.8% (95% confidence interval, 18.6 to 38.9%). Medications containing Glycyrrhizae radix for chronic kidney disease or loop diuretics were the most common UWC Kampo medications. Compared with patients who did not take any UWC Kampo medications, patients who did take such medications used aconite compositions more frequently (*p* = 0.04) and were more likely to have uncontrolled hypertension (*p* = 0.02) and chronic kidney disease (*p* <  0.001). In a multivariable analysis, no predictive factors for the use of UWC Kampo medications were identified.

**Conclusions:**

Approximately one-fourth of the elderly patients taking any prescribed Kampo medications took at least one UWC Kampo medication, although the association between UWC Kampo medications and adverse events remains unclear. When physicians prescribe medications containing Glycyrrhizae radix to elderly patients, chronic kidney disease and the use of loop diuretics should be evaluated.

## Background

Kampo medicine is a form of traditional Japanese herbal medicine. It was introduced to Japan from China 1500 years ago and was developed into a unique form of Japanese medicine over a long period in Japanese practice and culture [1–3]. Therefore, Kampo medicine is different from traditional Chinese and Korean medicines [3]. In Japan, Kampo medicines are integrated into the national health care system; 148 Kampo extract formulations and 187 types of crude drugs are approved by the Ministry of Health, Labour and Welfare and are used under the national health insurance programme [4, 5]. Kampo medications are available over the counter (OTC) and are the most frequently used complementary and alternative medicine therapies in Japan according to surveys of Japanese physicians [6–9].

Despite the common prescription of Kampo medications by Japanese physicians, there are no standard education programmes in medical schools and few postgraduate education programmes on Kampo medicines in Japan [3, 10, 11]. Given that a recent Japanese study reported some concerns regarding patient safety in relation to Kampo medications [4], it is important to ascertain the safety of the Kampo medications prescribed by physicians. Furthermore, given that adverse drug events are common in elderly patients due to multi-morbidity and polypharmacy [12–15], the appropriate use of Kampo medications by elderly patients is particularly important. Nonetheless, to our knowledge, no studies have investigated the appropriateness of the Kampo medications prescribed by physicians as part of typical care in Japan. Thus, our aim was to determine the prevalence of and the factors associated with the use of potentially inappropriate Kampo medications among elderly patients who take any prescribed Kampo medication.

## Methods

### Study design and location

The National Hospital Organization Tochigi Medical Center is a 350-bed community hospital in the Tochigi Prefecture of Japan. The centre is one of the two largest acute care hospitals in the area and serves approximately 0.5 million individuals. At our hospital, the internists mainly practise Western medicine but occasionally use Kampo medicine, depending on the patients’ and physicians’ preferences. No physicians receive formal training in Kampo medicine after graduation from a medical university. The aim of this study was to determine the prevalence and characteristics of elderly patients who were prescribed potentially inappropriate Kampo medications. A retrospective, single-centre, cross-sectional study was conducted using the electronic medical records of National Hospital Organization Tochigi Medical Center. The primary outcome was the proportion of patients who were prescribed at least one potentially inappropriate Kampo medication to be used with caution (hereafter denoted as UWC Kampo medications) among elderly ambulatory patients who were regularly prescribed any Kampo medications. We also evaluated the factors associated with the UWC Kampo medications. This study was approved by the Medical Ethical Committee of National Hospital Organization Tochigi Medical Center (No. 29–10) and was conducted in accordance with the Declaration of Helsinki. The need for individual informed consent was formally waived by the Medical Ethical Committee of National Hospital Organization Tochigi Medical Center because the study used data collected from medical records and the patients were not contacted.

### Participants and inclusion criteria

All consecutive ambulatory patients aged 65 years or older who had appointments with internal medicine physicians from January 1, 2015, to March 31, 2015, were retrospectively screened. This study included only patients who had 3 or more visits to internal medicine physicians within a year before the index visit and who were prescribed at least one Kampo medication on two or more consecutive visits. Over-the-counter Kampo medications were excluded. Patients with missing data regarding medications prescribed at other hospitals were excluded. During the study period, 1035 ambulatory patients aged 65 years or older were identified. Nine hundred fifty-five patients were excluded for a variety of reasons (895 for a lack of regular use of Kampo medications and 60 for missing data on medications prescribed at other hospitals). Thus, a total of 80 patients were included in the final analysis.

### Data collection

The data were collected using the electronic medical records of National Hospital Organization Tochigi Medical Center. All available electronic medical records from September 2014 to the time of the index visit were reviewed. Information regarding age, gender, past medical history, the Charlson Comorbidity Index (CCI) [16], office blood pressure, non-Kampo medications, and Kampo medications was retrieved. All prescriptions from our hospital were covered by the hospital database, but prescriptions from other hospitals were not covered. Therefore, prescriptions from other hospitals were identified based on medical record documentation by physicians in our hospital. The non-Kampo medications included oral medications, inhalers, injections, and as-needed medications. However, eye drops, intranasal infusers, OTC drugs, and topical medications were excluded, along with medications that were indicated for apparent transient disease and those that were not prescribed on 2 or more consecutive visits. For example, antitussive medications for the common cold and antibiotic agents for pharyngitis were not included. The numbers of non-Kampo and Kampo medications were counted separately.

### Outcome measures

The primary outcome was the proportion of elderly patients who took any UWC Kampo medications among those who took at least one prescribed Kampo medication. We defined UWC Kampo medications based on the 2015 traditional Chinese medicine Japan Geriatrics Society guidelines [17]. The following five criteria were used: (1) aconite composition use among patients with uncontrolled hypertension or tachyarrhythmia; (2) Glycyrrhizae radix composition use among patients with chronic kidney disease (CKD) or loop diuretic use; (3) ephedra composition use among patients with uncontrolled hypertension, ischaemic heart disease, or dysuria; (4) Scutellaria baicalensis composition use among patients with interferon use or liver cirrhosis; and (5) long-term use of gardenia composition (more than a few years). The representative Kampo medications that included these components and were identified in this study are shown in Table 1. Uncontrolled hypertension was defined as office systolic blood pressure ≥ 140 mmHg and/or diastolic blood pressure ≥ 90 mmHg [18–20]. If office blood pressure was not documented in medical records, hypertension was judged to be uncontrolled for patients whose antihypertensive medications were increased over three or more consecutive physician visits. Otherwise, hypertension was judged to be controlled. Tachyarrhythmia was defined as any arrhythmia that was controlled by antiarrhythmic medications. Only patients who were regularly prescribed medications for urinary problems were considered to have dysuria [21–23]. Long-term use of gardenia composition was defined as the use of gardenia for more than 3 years because the term “a few years”, which is used in the 2015 Japan Geriatrics Society guidelines [17], is ambiguous.

**Table 1:** Kampo medications containing aconite, Glycyrrhizae radix, ephedra, Scutellaria baicalensis or gardenia that were prescribed for 80 ambulatory elderly patients

Ingredient	Kampo medications
Aconite	Hachimijiogan, Goshajinkigan
Glycyrrhizae radix	Yokukansan, Shakuyakukanzoto, Bakumondoto, Kakkonto, Rikkunshito, Keishikashakuyakuto, Shakanzoto, Shoseiryuto, Ryokeijutsukanto, Junchoto, Hochuekkito, Kakkontokasenkyushini, Hangeshashinto, Saikokeishikankyoto, Shokenchuto, Kososan, Nijutsuto, Saireito, Bouiogito
Ephedra	Kakkonto, Shoseiryuto, Kakkontokasenkyushini
Scutellaria baicalensis	Junchoto, Hangeshashinto, Saikokeishikankyoto, Nijutsuto, Unseiin, Saireito
Gardenia	Unseiin

### Statistical analysis

The sample size was determined based on the following information. The interval between consecutive regular visits for most of the ambulatory patients at our hospital was a few months. Therefore, the patients who attended at least one physician visit from January 1, 2015, to March 31, 2015, were screened. The characteristics of the included patients were described using descriptive statistics. The primary outcome was the proportion of patients who were prescribed any UWC Kampo medication. The 95% confidence interval (CI) was calculated for this outcome. To compare the patient characteristics of patients who were and were not prescribed any UWC Kampo medications, Fisher’s exact tests were used for categorical variables, and Student’s t-tests were used for continuous variables. To identify the determinants of the prescription of UWC Kampo medications, multivariable analysis using binary logistic regression was also conducted. The following variables were included in the logistic regression model: age, gender, CCI, number of non-Kampo medications used, and the use of multiple Kampo medications. These analyses were conducted using IBM SPSS Statistics Base version 21.0 (IBM Corporation, Nihonbashi, Tokyo, Japan) or Excel statistical software package version 2.11 (Bellcurve for Excel; Social Survey Research Information Co., Ltd., Tokyo, Japan). The threshold for statistical significance was *p* <  0.05.

## Results

The characteristics of the included patients are presented in Table 2. Among the 80 patients, the mean age was 76.7 years, 45 (56.2%) were female, the mean CCI was 1.7, the median number of non-Kampo medications used was 5.0, and the median number of Kampo medications used was 1.0. The numbers of patients who took prescribed Kampo medications containing aconite, Glycyrrhizae radix, ephedra, Scutellaria baicalensis, and gardenia were 8 (10.0%), 57 (71.3%), 10 (12.5%), 6 (7.5%), and 1 (1.3%), respectively.

**Table 2:** Characteristics of the elderly ambulatory patients and the comparison of patients who were prescribed any potentially inappropriate Kampo medications to be used with caution and those who were not

		Prescription of any potentially inappropriate Kampo medications^a^		*p*-value^b^
	Total *n* = 80	Yes *n* = 23	No *n* = 57	
Age, mean ± SD	76.7 ± 8.1	78.1 ± 8.3	76.2 ± 8.0	0.34
Female, *n* (%)	45 (56.2)	17 (73.9)	28 (49.1)	0.05
Charlson Comorbidity Index, mean ± SD	1.7 ± 1.8	1.8 ± 2.0	1.6 ± 1.8	0.62
Non-Kampo medications^c^				
Total number				
Median (IQR)	5.0 (3.0–7.3)	6.0 (4.0–8.5)	5.0 (3.0–7.0)	0.80
Mean ± SD	5.6 ± 3.0	6.4 ± 3.1	5.3 ± 3.0	0.16
Five or more medications, *n* (%)	46 (57.5)	14 (60.9)	32 (56.1)	0.80
Kampo medications				
Total number				
Median (IQR)	1.0 (1.0–1.0)	1.0 (1.0–2.0)	1.0 (1.0–1.0)	NA
Mean ± SD	1.2 ± 0.4	1.3 ± 0.5	1.1 ± 0.3	0.05
Two or more medications, *n* (%)	14 (17.5)	7 (30.4)	7 (12.3)	0.10
Aconite composition, *n* (%)	8 (10.0)	5 (21.7)	3 (5.3)	0.04
Glycyrrhizae radix composition, *n* (%)	57 (71.3)	19 (82.6)	38 (66.7)	0.18
Ephedra composition, *n* (%)	10 (12.5)	3 (13.0)	7 (12.3)	1.00
Scutellaria baicalensis composition, *n* (%)	6 (7.5)	2 (8.7)	4 (7.0)	1.00
Gardenia composition, *n* (%)	1 (1.3)	1 (4.4)	0 (0.0)	0.28
Specific medications, *n* (%)				
Three or more antihypertensive drugs	14 (17.5)	7 (30.4)	7 (12.3)	0.10
Loop diuretics	6 (7.5)	1 (4.4)	5 (8.8)	0.67
Dysuria medications	9 (11.3)	4 (17.4)	5 (8.8)	0.43
Interferon	0 (0.0)	0 (0.0)	0 (0.0)	NA
Uncontrolled hypertension^d^, *n* (%)	20 (25.0)	10 (43.5)	10 (17.5)	0.02
Past medical history, *n* (%)				
Tachyarrhythmia^e^	3 (3.8)	0 (0.0)	3 (5.3)	0.55
Dementia	13 (16.3)	4 (17.4)	9 (15.8)	1.00
Ischaemic heart disease	7 (8.8)	1 (4.4)	6 (10.5)	0.45
Chronic kidney disease	25 (31.3)	18 (78.3)	7 (12.3)	< 0.001
Liver cirrhosis	4 (5.0)	2 (8.7)	2 (3.5)	0.57
Hypertension	50 (62.5)	17 (73.9)	33 (57.9)	0.21

^a^Defined based on the 2015 Japan Geriatrics Society guidelines

^b^Fisher’s exact tests or Student’s t-tests were used for comparisons between patients who were and were not prescribed any potentially inappropriate Kampo medications to be used with caution. The threshold for statistical significance was set at *p* < 0.05

^c^Excluding Kampo medications

^d^Uncontrolled hypertension was defined as office systolic blood pressure ≥ 140 mmHg and/or diastolic blood pressure ≥ 90 mmHg

^e^Tachyarrhythmia was defined as any arrhythmia that was controlled with antiarrhythmic medication

The proportion of patients who were prescribed any UWC Kampo medications was 28.8% (95% CI, 18.6 to 38.9%; Table 3). Compared with patients who did not take any UWC Kampo medications, patients who did take such medications used aconite composition more frequently (*p* = 0.04) and were more likely to have uncontrolled hypertension (*p* = 0.02) and CKD (*p* < 0.001). The proportion of female gender and use of multiple Kampo medications tended to be higher in patients who took any UWC Kampo medications than in patients who did not, although these associations were not statistically significant. Among the UWC Kampo medications, medications containing Glycyrrhizae radix prescribed for patients with CKD or loop diuretic users were the most commonly used (*n* = 16, 20.0%), followed by those containing aconite prescribed for patients with uncontrolled hypertension or tachyarrhythmia (*n* = 5, 6.3%).

**Table 3:** Prevalence of the use potentially inappropriate Kampo medications to be used with caution^a^ in elderly patients taking any prescribed Kampo medications

Characteristic	Total *n* = 80
Any potentially inappropriate Kampo medications to be used with caution, *n* (%)	23 (28.8)
Aconite for uncontrolled hypertension^b^ or tachyarrhythmia^c^ patients, *n* (%)	5 (6.3)
Glycyrrhizae radix for CKD patients or loop diuretic users, *n* (%)	16 (20.0)
Ephedra for uncontrolled hypertension^b^, ischaemic heart disease, or dysuria patients, *n* (%)	1 (1.3)
Scutellaria baicalensis for interferon users or liver cirrhosis patients, *n* (%)	0 (0.0)
Long-term use^d^ of gardenia, *n* (%)	1 (1.3)

^a^Defined based on the 2015 Japan Geriatrics Society guidelines

^b^Uncontrolled hypertension was defined as office systolic blood pressure ≥ 140 mmHg and/or diastolic blood pressure ≥ 90 mmHg

^c^Tachyarrhythmia was defined as any arrhythmia that was controlled with antiarrhythmic medications

^d^Long-term use was defined as use for more than 3 years

Table 4 presents a summary of the results of the multivariable analysis using logistic regression to identify predictors of the use of UWC Kampo medications. Among the five included variables (i.e., age, gender, CCI, number of non-Kampo medications, and multiple Kampo medications), no independent predictive factors for the use of UWC Kampo medications were identified.

**Table 4:** Summary results of multivariable logistic regression analyses of predictive factors of the use of potentially inappropriate Kampo medications to be used with caution^a^

Variable	Odds ratio (95% confidence interval)			
	Unadjusted	*p*-value^b^	Adjusted^c^	*p*-value^b^
Age	1.03 (0.97–1.09)	0.34	1.02 (0.96–1.09)	0.50
Female	2.93 (1.01–8.52)	0.047	2.95 (0.95–9.13)	0.06
Charlson Comorbidity Index	1.07 (0.83–1.38)	0.61	1.10 (0.82–1.48)	0.52
Number of non-Kampo medications^d^	1.12 (0.96–1.32)	0.16	1.09 (0.92–1.30)	0.98
Multiple Kampo medications	3.13 (0.95–10.27)	0.06	3.47 (0.98–12.26)	0.05

^a^Defined based on the 2015 Japan Geriatrics Society guidelines

^b^Statistical significance was evaluated at *p* < 0.05

^c^The following variables were adjusted: age, gender, CCI, number of non-Kampo medications, and multiple Kampo medications

^d^Excluding Kampo medications

## Discussion

This study found that approximately one-fourth of elderly patients taking prescribed Kampo medications took UWC Kampo medications. Most of the UWC Kampo medications were medications containing Glycyrrhizae radix prescribed for CKD patients or loop diuretic users and medications containing aconite prescribed for patients with uncontrolled hypertension or tachyarrhythmia. Compared with patients who did not take any UWC Kampo medications, those who did used aconite composition more frequently (*p* = 0.04) and were more likely to have uncontrolled hypertension (*p* = 0.02) and CKD (*p* < 0.001).

To our knowledge, the present study is the first to evaluate the appropriateness of Kampo medications prescribed by physicians for elderly patients in Japan. Because no data have been published regarding the appropriateness of Kampo medications prescribed by physicians, it is unknown whether the present results are consistent with data from other hospitals in Japan. However, the prevalence of the use of UWC Kampo medications in this study is similar to the reported prevalences of potential adverse herb-drug interactions among patients using herbal medicines in previous studies [24–27]. Furthermore, the prevalence of the use of potentially inappropriate Western medications among community-dwelling elderly patients ranges from 20 to 40% [28, 29]. Therefore, the prevalence of the use of UWC Kampo medications observed in this study might reflect the true prevalence of their use among elderly patients in Japan. Considering the common use of prescribed Kampo medications in Japan [6–9, 30–33], it is surprising that few studies have evaluated the appropriateness of Kampo medication use. Further studies are warranted to determine the prevalence of the use of UWC Kampo medications among elderly patients at other hospitals and in other settings in Japan, and efforts are needed to improve the appropriateness of Kampo medication use among elderly patients. In this study, most of the UWC Kampo medications were medications containing Glycyrrhizae radix prescribed for CKD patients or loop diuretic users and medications containing aconite prescribed for patients with uncontrolled hypertension or tachyarrhythmia. These findings imply that physicians should be cautious when they use Kampo medications containing Glycyrrhizae radix or aconite for elderly patients.

To evaluate the appropriateness of Kampo medication use, we used the definitions of UWC Kampo medications based on the 2015 Japan Geriatrics Society guidelines on traditional Chinese medicine [17]. The three definitions of UWC Kampo medications regarding aconite, Glycyrrhizae radix, and ephedra compositions correspond to contraindications or precautions based on another authoritative resource [34] and review [35]. However, all five definitions applied in this study are based on low-quality studies, such as a case reports and case series [17, 36], because high-quality clinical data regarding the safety of herbal medicines are lacking [35, 36]. Therefore, it is unclear whether UWC Kampo medications are truly associated with worse patient outcomes. Further studies are needed to evaluate whether UWC Kampo medications increase the risk of adverse events.

In the multivariable analysis of this study, the use of multiple Kampo medications was not significantly associated with any use of UWC Kampo medications. However, given that both polypharmacy and the number of medications are strong independent risk factors for the use of potentially inappropriate medications (PIMs) among elderly patients in studies of Western medications [28, 37], and multiple Kampo medications should be used with caution. Considering the limitations of this study, e.g., the small sample size and single-centre design, further studies are warranted to evaluate this association between the use of multiple Kampo medications and the use of UWC Kampo medications.

### Limitations

Our results should be interpreted in the context of several limitations. First, the study used a retrospective design. Second, the information on prescriptions from other hospitals might be inaccurate because prescriptions from other hospitals were determined according to the documentation of such prescriptions in patient medical records by physicians at our hospital. Third, the indications for Kampo medications were not evaluated. Therefore, the intended uses of these medications were unclear. Fourth, the use of OTC Kampo medications was not investigated. Furthermore, the transient use of Kampo medications was not considered this study. Fifth, adherence to medication regimens was not assessed. Sixth, we did not evaluate the severity of comorbidities, which might affect the risk of the use of UWC Kampo medications. Seventh, this study was limited to a single centre and included a small sample size. Therefore, these findings should be confirmed by multicentre or population-level studies in the future.

## Conclusions

Approximately one-fourth of the elderly patients who took prescribed Kampo medications also took UWC Kampo medications. Medications containing Glycyrrhizae radix prescribed to patients with CKD or loop diuretic users were the most common UWC Kampo medications. Before medications containing Glycyrrhizae radix are prescribed for elderly patients, CKD and loop diuretic use should be checked. These findings need to be confirmed in other settings in Japan.

## Abbreviations

CCI: Charlson Comorbidity Index
CI: Confidence interval
CKD: Chronic kidney disease
IQR: Interquartile range
NA: Not applicable
OR: Odds ratio
OTC: Over the counter
PIM: Potentially inappropriate medication
SD: Standard deviation
UWC Kampo medication: Potentially inappropriate Kampo medication to be used with caution
